# The Antianginal Drug Perhexiline Displays Cytotoxicity against Colorectal Cancer Cells In Vitro: A Potential for Drug Repurposing

**DOI:** 10.3390/cancers14041043

**Published:** 2022-02-18

**Authors:** Bimala Dhakal, Celine Man Ying Li, Runhao Li, Kenny Yeo, Josephine A. Wright, Krystyna A. Gieniec, Laura Vrbanac, Tarik Sammour, Matthew Lawrence, Michelle Thomas, Mark Lewis, Joanne Perry, Daniel L. Worthley, Susan L. Woods, Paul Drew, Benedetta C. Sallustio, Eric Smith, John D. Horowitz, Guy J. Maddern, Giovanni Licari, Kevin Fenix

**Affiliations:** 1 Department of Surgery, Adelaide Medical School, The University of Adelaide, Adelaide, SA 5005, Australia; bimala.dhakal@adelaide.edu.au (B.D.); manying.li@adelaide.edu.au (C.M.Y.L.); runhao.li@adelaide.edu.au (R.L.); kenny.yeo@adelaide.edu.au (K.Y.); paul.drew@adelaide.edu.au (P.D.); eric.smith@adelaide.edu.au (E.S.); guy.maddern@adelaide.edu.au (G.J.M.); 2 The Basil Hetzel Institute for Translational Health Research, The Queen Elizabeth Hospital, The University of Adelaide, Woodville, SA 5011, Australia; benedetta.sallustio@sa.gov.au (B.C.S.); john.horowitz@adelaide.edu.au (J.D.H.); 3 Medical Oncology, The Queen Elizabeth Hospital, Woodville, SA 5011, Australia; 4 Precision Medicine, South Australian Health and Medical Research Institute, Adelaide, SA 5005, Australia; josephine.wright@sahmri.com (J.A.W.); krystna.gienic@adelaide.edu.au (K.A.G.); laura.vrbanac@adelaide.edu.au (L.V.); sammourt@colorectalsa.com.au (T.S.); dan@colonoscopyclinic.com.au (D.L.W.); susan.woods@adelaide.edu.au (S.L.W.); 5 Department of Medical Specialties, Adelaide Medical School, The University of Adelaide, Adelaide, SA 5005, Australia; 6 Colorectal Unit, Department of Surgery, Royal Adelaide Hospital, Adelaide, SA 5005, Australia; lawrencemj@colorectalsa.com.au (M.L.); michelle.thomas@sa.gov.au (M.T.); mark.lewis2@sa.gov.au (M.L.); joanne.perry@sa.gov.au (J.P.); 7 Discipline of Pharmacology, Adelaide Medical School, The University of Adelaide, Adelaide, SA 5005, Australia

**Keywords:** perhexiline, colorectal cancer, patient-derived organoids, perhexiline enantiomers, anti-tumour agents

## Abstract

**Simple Summary:**

Cancer cells frequently have an altered metabolism to support their increased proliferative and invasive activity. Perhexiline, a drug used to treat some cardiovascular diseases, inhibits some of the reported changes in the metabolism of cancer cells. We show that treatment with this drug either as a racemate or its enantiomers can kill colorectal cancer cells. The drug has been used clinically for a long time and has potential to be repurposed for use in the management of colorectal cancer.

**Abstract:**

Colorectal cancer (CRC) is the second leading cause of cancer-related death worldwide. Perhexiline, a prophylactic anti-anginal drug, has been reported to have anti-tumour effects both in vitro and in vivo. Perhexiline as used clinically is a 50:50 racemic mixture ((R)-P) of (−) and (+) enantiomers. It is not known if the enantiomers differ in terms of their effects on cancer. In this study, we examined the cytotoxic capacity of perhexiline and its enantiomers ((−)-P and (+)-P) on CRC cell lines, grown as monolayers or spheroids, and patient-derived organoids. Treatment of CRC cell lines with (R)-P, (−)-P or (+)-P reduced cell viability, with IC_50_ values of ~4 µM. Treatment was associated with an increase in annexin V staining and caspase 3/7 activation, indicating apoptosis induction. Caspase 3/7 activation and loss of structural integrity were also observed in CRC cell lines grown as spheroids. Drug treatment at clinically relevant concentrations significantly reduced the viability of patient-derived CRC organoids. Given these in vitro findings, perhexiline, as a racemic mixture or its enantiomers, warrants further investigation as a repurposed drug for use in the management of CRC.

## 1. Introduction

Colorectal cancer (CRC) is one of the most commonly diagnosed cancers worldwide, and is a leading cause of cancer-related death [1]. Despite improvements in prevention and treatment strategies, the global burden of CRC is anticipated to rise by 60% with 2.2 million new cases and 1.1 million deaths annually by 2030 [2]. About 50% of patients with CRC either present with, or will develop, metastatic disease, which, despite an increasing range of treatment options, remains a relatively lethal disease with a five-year survival rate of ~15%. This highlights the need to continue searching for new treatment strategies [3].

Alterations in metabolism are common in cancer cells, providing the increased energy and materials for functions characteristic of cancer, such as unlimited proliferation, tissue invasion and metastasis. One of the many metabolic changes reported in cancer cells is in the biosynthesis and oxidation of fatty acids. Fatty acid oxidation (FAO) results in the production of ATP and NADPH. In many cancers FAO is the major source of ATP for tumour growth, and NADPH helps protect cancer cells against oxidative stress and cell death. In addition, FAO can promote metastasis by inducing epithelial–mesenchymal transitions in cancer stem cells [4]. Alterations in FAO are an important change in cancer metabolism.

Perhexiline, 2-(2,2-dicyclohexylethyl), originally developed as an antianginal drug in the 1970s, is used clinically to treat a number of cardiac conditions, including angina, aortic stenosis and heart failures [5,6,7]. A major effect of perhexiline is reduction in fatty acid metabolism through the inhibition of carnitine palmitoyltransferase-1 (CPT-1), an enzyme responsible for mitochondrial uptake of long-chain fatty acids and the rate-limiting step in their mitochondrial metabolism [5]. Perhexiline, by disrupting FAO, could be expected to alter the growth of cancer cells. There have been few studies investigating the effect of perhexiline on the viability of cancer cells and none focused solely on CRC.

Given the importance of fatty acid metabolism in cancer cells, we hypothesised that perhexiline, by altering FAO, would reduce the viability of CRC cells. Pharmaceutical preparations of perhexiline consist of a racemic mixture (R) of the (+) and (–) enantiomers [5]. The enantiomers have been shown to differ slightly in pharmacokinetic properties in patients [8,9], but it is not known if they differ in their effect on the viability of cancer cells. Here we report for the first time that perhexiline and its enantiomers equally reduce the viability of CRC cell lines grown as monolayers or spheroids and patient-derived CRC organoids in vitro.

## 2. Materials and Methods

### 2.1. Reagents

Perhexiline maleate salt, (R)-P (≥95% purity by HPLC; CAS no: 6724-53-4) was purchased from (Sigma-Aldrich, St. Louis, MO, USA). The individual enantiomers (−)-P and (+)-P were prepared using previously reported methods [9]. The drugs were prepared in incubation medium with a final concentration (*v*/*v*) of 1% ethanol (Sigma-Aldrich, USA). The stock concentrations of drugs were quality checked using mass spectrometry.

### 2.2. Cell Lines and Culture

The CRC cell lines (SW480, SW620, HCT116, HT29 and COLO205) and human foreskin fibroblasts (HFF) were obtained from the American Type Culture Collection (ATCC, USA). SW480, SW620, HCT116 and HFF were maintained in culture media consisting of DMEM (Life Technologies, Carlsbad, CA, USA) supplemented with 10% heat-inactivated fetal bovine serum (Sigma-Aldrich, USA), 200 U/mL penicillin, 200 µg/mL streptomycin and 200 mM GlutaMAX Supplement (Life Technologies, USA). HT29 and COLO205 were maintained in RPMI medium (Life Technologies, USA) with similar supplements used for DMEM media and incubated at 37 °C with 5% CO_2_ in air. All cells were mycoplasma-free (MycoAlert mycoplasma detection kit; Lonza, Switzerland).

### 2.3. Patient-Derived Organoids

Patient-derived organoids were cultured as described previously [10]. Tumour samples were first minced and then enzymatically digested in organoid digestion media consisting of DMEM containing 67.5 U/mL collagenase IV (CLS-4 Worthington), 0.23 U/mL dispase (Life Technologies, USA), 100 U/mL penicillin and 100 mg/mL streptomycin (all from Life Technologies, USA), 8–20 U/mL hyaluronidase and 50 Kunitz units/mL DNase Type I (Sigma Aldrich, USA) for 30–60 min in a water bath at 37 °C. Organoids were embedded in 50 μL matrigel domes and cultured in low (5–6%)-oxygen conditions in CRC organoid media containing advanced DMEM/F12, 10 mM Hepes, 1X GlutaMAX Supplement, 10 mg/L gentamicin, 1X antibiotic-antimycotic, 2X B27 (all from Life Technologies, USA), 500 nM A83–01 (Tocris Bioscience, Bristol UK), 50 ng/mL hEGF, 1 nM [Leu15]-gastrin 1, 1 mM N-acetyl-L cysteine, 5 µM SB202190, 10 µM SB431542, and 10 µM Y27632 (all from Sigma-Aldrich, USA). The CRC organoid media was changed twice weekly, with growth monitored until passaging was required. Organoids were passaged upon reaching 100–200 mm in diameter by digestion with TrypLE (Life Technologies, USA) at 37 °C followed by trituration with a pipette until the desired number of cells for cytotoxicity assay was obtained. Organoids derived from normal liver tissue were prepared and cultured similarly to CRC organoids but using media containing advanced DMEM/F12, 1X N2, 1X B27 (all from Life Technologies), 1.25 mM N-acetylcysteine, 10 nM gastrin, 50 ng/mL EGF, 10 µM Y27632, 10 mM nicotinamide (all from Sigma-Aldrich), 10% Rspo2 conditioned medium, 100ng/mL FGF10, 25ng/mL HGF (all from Peprotech, Rocky Hill, CT, USA, 5 µM A83-01 and 10 µM forskolin (all from Tocris Bioscience, Bristol, UK).

### 2.4. Crystal Violet Viability Assay

Cell growth was determined by crystal violet assay, as described previously [11]. Briefly, 3 × 10^3^ SW480, SW620, HT29 or COLO205 cells, or 1 × 10^3^ HCT116 cells, were seeded into 96-well plates and cultured for 24 h. Cells were treated for 72 h with either 1, 2, 4, 6, 8 or 10 µM of ®-P, (−)-P or (+)-P in a final concentration of 1% (*v*/*v*) ethanol. Following treatment, the cells were fixed with 10% neutral buffered formalin for 30 min, stained with 1% (*w*/*v*) crystal violet (Sigma-Aldrich, USA) in 2% ethanol for 10 min, washed eight times in running distilled water and air-dried. Crystal violet was eluted using 10% acetic acid with gentle rocking of the plates for 2 h at room temperature. Absorbance of the eluent was measured at 595 nm using a FLUOstar Optima microplate reader (BMG Labtech, Ortenburg, Germany). The average absorbance of the wells without cells was subtracted from the absorbance of each of the wells containing cells and the data were expressed as the mean absorbance relative to that of the vehicle control treated cells.

### 2.5. Apoptosis Assay by Annexin V/Propidium Iodide Staining

SW620 and HT29 cells were seeded at 0.25 × 10^6^ cells per well in six-well plates and incubated for 24 h. Cells were washed with Dulbecco’s phosphate buffered saline (DPBS) (Life Technologies, USA) to remove non-viable cells and then treated for 48 h with either 0, 4, 6, 8 or 10 µM of (−)-P, (+)-P or (R)-P. Culture media containing the non-adherent cells were collected. Adherent cells were then washed three times with DPBS, collecting each wash, and incubated with 0.25% trypsin-EDTA (Life Technologies, USA) at 37 °C for 4 min, after which trypsin was inactivated with complete cell culture media containing the supplements previously listed. The cells were harvested by pooling the non-adherent and adherent cells, centrifuging at 400 g for 5 min at 4 °C, removing the supernatant and resuspending the cell pellets in DPBS. Then, 0.5 × 10^5^ cells were stained using the APC Annexin V Apoptosis Detection Kit with PI (Biolegend, San Diego, CA, USA) as per the manufacturer’s instructions. Cells were analysed using a FACS Canto II (BD Biosciences), gating out debris and doublets, and acquiring at least 50,000 single cell events per sample. Quantification of early apoptotic (annexin V-positive) cells was performed using FlowJo v10.4.1 (FlowJo, Ashland, USA).

### 2.6. Caspase 3/7 Activation Assay

Caspase 3/7 activation in response to drug treatment was measured in CRC cell lines grown as monolayers or as spheroids. For monolayers, 3 × 10^3^ of SW620 or HT 29, or 1 × 10^3^ HCT116 cells were seeded in a 96-well plate and incubated overnight. The cells were then treated for 72 h with either 1, 2, 4, 6, 8 or 10 µM of (−)-P, (+)-P or (R)-P in media containing 1 µM CellEvent Caspase-3/7 Green Detection Reagent (Thermo Fisher Scientific, Waltham, MA, USA). For spheroids, 1 × 10^4^ HT29 or HCT116 cells were seeded into a round bottom ultra-low attachment plate (Corning, New York, NY, USA) and incubated for 96 h to form 3-D spheroids. The spheroids were then treated for 96 h with either 1, 2, 4, 6, 8 or 10 µM of (−)-P, (+)-P or (R)-P containing 1 µM Caspase-3/7 Green reagent. The activation of caspase 3/7 was monitored for 72 h and the data were analysed using the Incucyte S3 Live Analysis System software.

### 2.7. AlamarBlue Viability Assay for Patient-Derived Organoids

Established patient-derived organoids were digested into single cells and 2×10^3^ cells were seeded in 10 µL Matrigel domes in 96-well white walled plates and cultured for 48 h. They were then treated for 6 days with either 0.625, 1.25, 2.5, 5 or 10 µM of (−)-P, (+)-P or (R)-P. Images were taken on day six to study the morphological differences between the groups. AlamarBlue HS Cell Viability Reagent (Thermo Fisher Scientific, USA) was added at 1/10 dilution followed by incubation for 4 h. Fluorescence intensity was measured with a 560 nm excitation and a 590 nm emission filter using a FLUOstar Optima microplate reader (BMG Labtech, Germany). The data were expressed as the mean percent viability relative to that of the vehicle control treated organoids.

### 2.8. Statistical Analysis

All the statistical analyses were performed using GraphPad Prism Version 9 (GraphPad Software, San Diego, CA, USA).

## 3. Results

### 3.1. Effect of Perhexiline and Its Enatiomers on CRC Monolayer Growth

**Figure 1:**
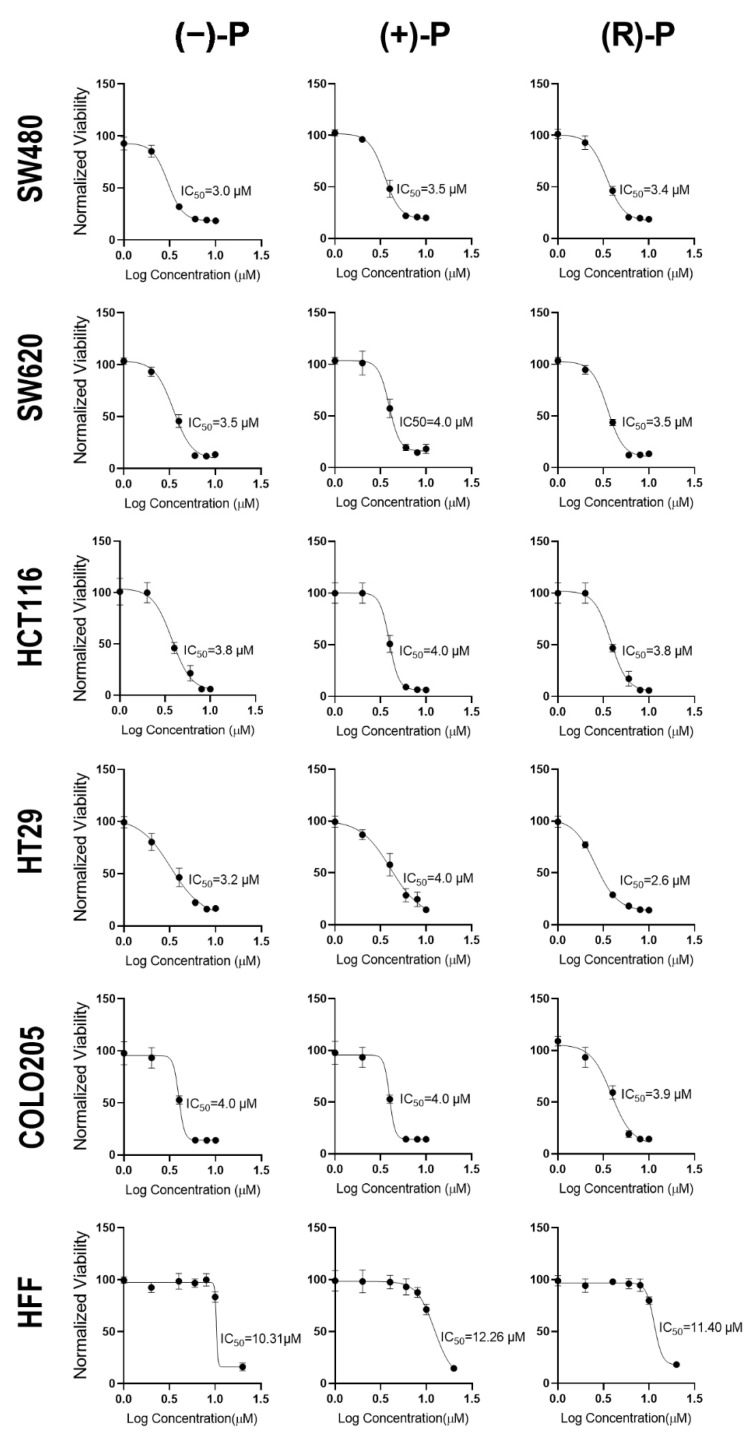
The IC_50_ for (−)-P, (+)-P and (R)-P in CRC and fibroblast cell lines. CRC cell lines and human foreskin fibroblasts (HFF) were treated with different concentrations of (−)-P, (+)-P and (R)-P and their viability was determined by crystal violet assay. Data are the mean ± standard deviation (SD) of five technical replicates from a representative experiment normalised to the vehicle control. Non-linear regression analysis for (−)-P, (+)-P and (R)-P was used to calculate IC_50_ values for each form of drug. Data are representative of three independent experiments.

To determine their ability to inhibit cell growth, CRC cell lines and human foreskin fibroblasts (HFF) were exposed to a range of concentrations of (−)-P, (+)-P and (R)-P, and the growth of the adherent monolayer was measured by crystal violet staining. Our results show that each of the compounds significantly reduced the growth of CRC cell lines at similar concentrations, with complete inhibition observed with 8 µM. In contrast, HFF were more tolerant, with complete inhibition of cell growth only observed at 20 µM (Figure 1). All five CRC cell lines tested had an IC_50_ of ~4 µM, while that for HFFs was ~11 µM (Table 1). These data, indicating selectivity of cell growth inhibition by perhexiline for cancer cells (relative to normal cells, as shown in our selectivity index Table 2) are consistent with previously reported results of a study on gastric cancers [12].

### 3.2. Effect of Perhexiline and Its Enantiomers on Annexin V Induction

**Figure 2:**
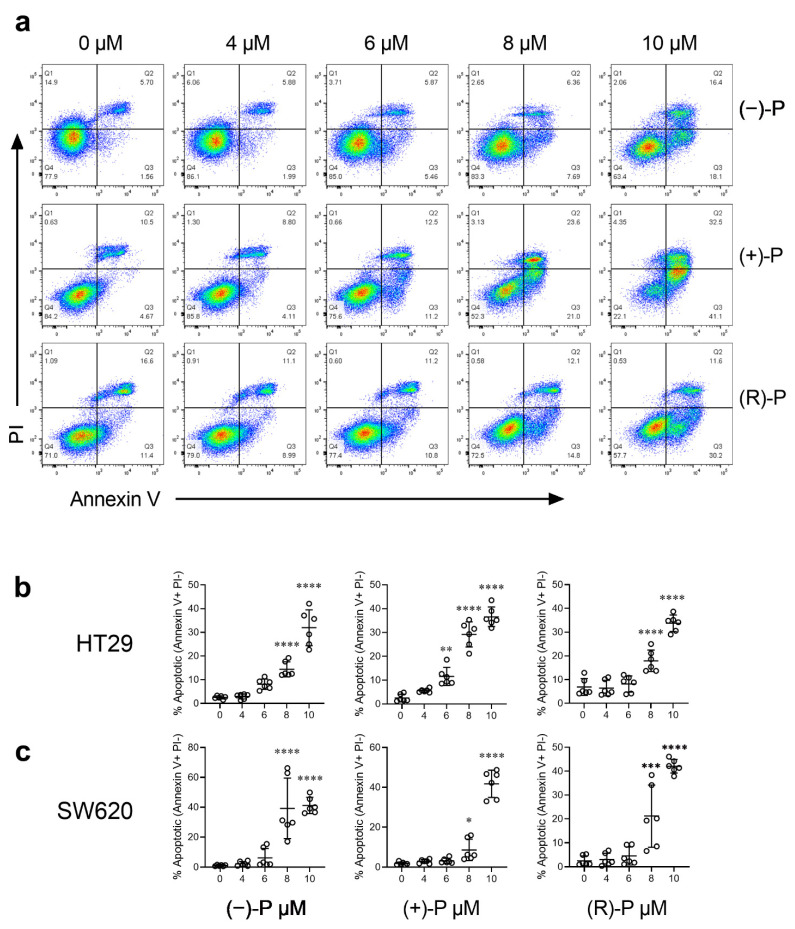
The effect of treatment with (−)-P, (+)-P, or (R)-P on Annexin V staining in CRC cell lines. HT29 or SW620 cells were treated with different concentrations of -P, (+)-P and (R)-P for 48 h, then stained with Annexin V/PI and analysed by flow cytometry. (**a**) Representative flow cytometry scatterplots, showing viable (Q4, double negative), early apoptotic (Q3, annexin V positive), late apoptotic (Q2, annexin V and PI positive) and necrotic (Q1, PI positive) HT29 cells. (**b**) The percentage of early apoptotic cells in HT29 cultures. (**c**) The percentage of early apoptotic cells in SW620 cultures. Results shown are mean ± SD of early apoptotic cells from technical replicates pooled from three independent experiments. * *p* ≤ 0.05, ** *p* ≤ 0.01, *** *p* ≤ 0.001, **** *p* ≤ 0.0001. One-way ANOVA with multiple comparisons test against vehicle (0 µM) control.

To determine if the CRC growth inhibition could be attributed to perhexiline-mediated apoptosis, HT29 or SW620 cells were treated with (−)-P, (+)-P and (R)-P for 48 h, stained using the Annexin V/PI staining kit and analysed by flow cytometry. Early apoptosis was specifically measured by gating for annexin V+ PI- populations (Q3, Figure 2a). Our results confirm that (−)-P, (+)-P, and (R)-P induced apoptosis at concentrations similar to the IC_50_s we had determined for the HT29 and SW620 cells (Figure 2b,c).

### 3.3. Kinetics of Apoptosis Induction by Perhexiline and Its Enantiomers

**Figure 3:**
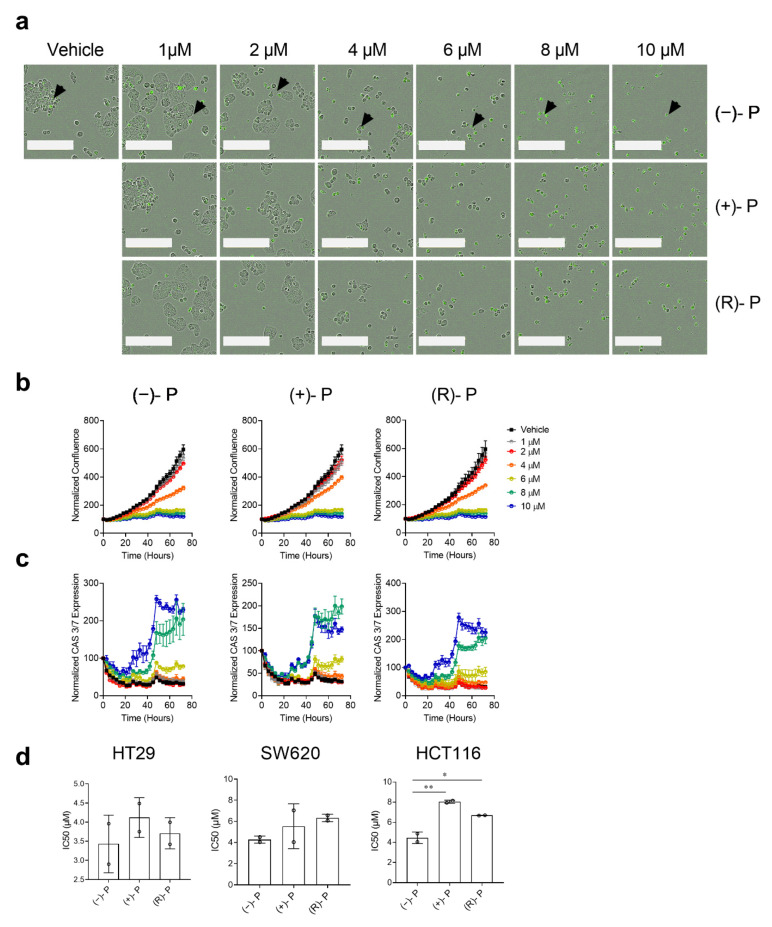
The kinetics of (−)-P-, (+)-P- and (R)-P-mediated apoptosis of CRC cells grown in monolayers. CRC cells were monitored for 72 h in the presence of (−)-P, (+)-P and (R)-P and Caspase-3/7 Green reagent using an Incucyte S3 Live Analysis System. (**a**) Representative images of HT29 cells 72 h post drug treatment. Caspase 3/7 positive cells are shown as green, indicated by the black arrows in (−)-P. The white bar represents 200 µm. (**b**) Cell growth curves measured by calculating the confluence (%) from time 0. (**c**) Caspase 3/7 kinetics curves measured by calculating CAS 3/7+ (green) cells normalized to time 0. Individual points represent the mean ± SD from three technical replicates in a single experiment. Data are representative of two independent experiments. (**d**) The IC_50_ values for the HT29, SW620 and HCT116 cell lines calculated and pooled from two independent experiments. Mean ± SD, * *p* ≤ 0.05, ** *p* ≤ 0.001. One-way ANOVA with a multiple comparisons test.

To understand the kinetics of apoptosis induction mediated by perhexiline on CRC cell lines, we treated HT29 cells grown as a monolayer with various concentrations of (−)-P, (+)-P and (R)-P and performed live-cell imaging to continuously monitor cell growth for 72 h in the presence of Caspase-3/7 Green reagent. Treatment with perhexiline compounds inhibited HT29 cell growth, with morphological changes, such as cellular shrinkage and blebbing and caspase 3/7 activation, becoming prominent at concentrations above 4 µM (Figure 3a). Cell growth, as measured by confluence, increased exponentially from approximately 24 h post seeding (Figure 3b). This was inhibited by concentrations of ≥ 4 µM of perhexiline or its enantiomers and coincided with an increase in caspase 3/7 induction which peaked at 40 h post treatment (Figure 3c). Similar caspase 3/7 induction was also detected in treated SW620 and HCT116 cells (Figure S1). There was no statistical difference between the IC50s for the (−)-P, (+)-P or (R)-P in cultures of either HT29 or SW620 cell lines. However, a small but statistically significant difference was observed in the HCT116 cell line, with (−)-P having a lower IC50 (4.5 µM) than (+)-P (8 µM) and (R)-P (6.7 µM) (Figure 3d).

### 3.4. Effect of Perhexiline and Its Enantiomers on CRC Spheroids

**Figure 4:**
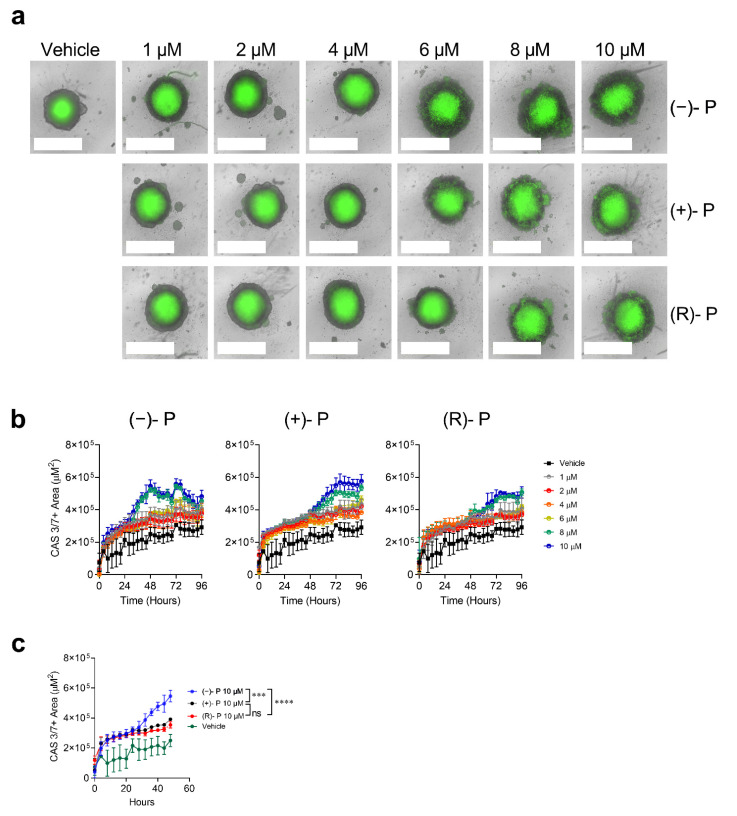
The kinetics of (−)-P-, (+)-P- and (R)-P-induced caspase 3/7 activation in HT29 cells grown as spheroids. HT29 spheroids were grown for 96 h, followed by live-cell imaging for a further 96 h in the presence of (−)-P, (+)-P and (R)-P and Caspase-3/7 Green reagent using an Incucyte S3 Live analysis system. (**a**) Representative images of HT29 spheroids 96 h post drug treatment. White bars represent 800 µm. (**b**) Caspase 3/7 kinetics curves generated by measuring the caspase 3/7 positive area within HT29 spheroids for (−)-P, (+)-P and (R)-P over 96 h. (**c**) Comparison of caspase 3/7 activation over 48 h after treatment with vehicle or 10 µM (−)-P, (+)-P and (R)-P. Individual points represent the mean ± SD from three technical replicates in a single experiment. *** *p* ≤ 0.001, **** *p* ≤ 0.0001. Two-way ANOVA with multiple comparisons test. Data are representative of two independent experiments.

Three-dimensional in vitro cultures are thought to better represent the behaviours of cancer cells in vivo, compared to 2D monolayers, particularly with respect to sensitivity to drugs, in part due to their ability to retain tumour-specific structures and cell sub-specializations [13,14]. Tumour spheroids tend to have higher resistance to drugs compared to monolayers [14]. To determine if perhexiline and its enantiomers are effective against 3D tumour spheroids, we grew HT29 cells in non-adherent conditions using ultralow attachment plates. In this setting, HT29 spheroids form a hypoxic inner core composed of cells undergoing apoptosis and an outer layer of proliferating cells responsible for spheroid growth [15]. The HT29 spheroids were treated with a range of concentrations of (−)-P, (+)-P or (R)-P. The perhexiline compounds increased caspase 3/7-mediated apoptosis. Loss of spheroid integrity, seen as a disruption of the clear boundaries and caspase 3/7 activation in the outer layer, was observed following treatment with (−)-P, (+)-P and (R)-P at ≥6 µM (Figure 4a). Whilst all forms of the drug were cytotoxic to the spheroids, as quantitated by caspase 3/7 activation, the kinetics showed that (−)-P acted more quickly than the (+)-P or (R)-P (Figure 4b). For example, at 10 µM, spheroid caspase 3/7 activation peaked within 48 h for (−)-P when compared to (+)-P and (R)-P, which peaked at around 72 h (Figure 4c, Supplementary Videos S1–S4). Similar results were observed in the HCT116 spheroids (Figure S2).

### 3.5. Effect of Perhexiline and Its Enantiomers on Patient-Derived CRC Organoids

In recent years patient-derived tumour organoids have emerged as a ‘gold standard’ in vitro model for studying drug sensitivity responses and have been increasingly used in high-throughput screening to design personalized treatment regimens for cancer patients [10,16]. Therefore, we measured the effect of perhexiline on patient-derived CRC organoids derived from primary and metastatic sites (Table 3).

**Figure 5:**
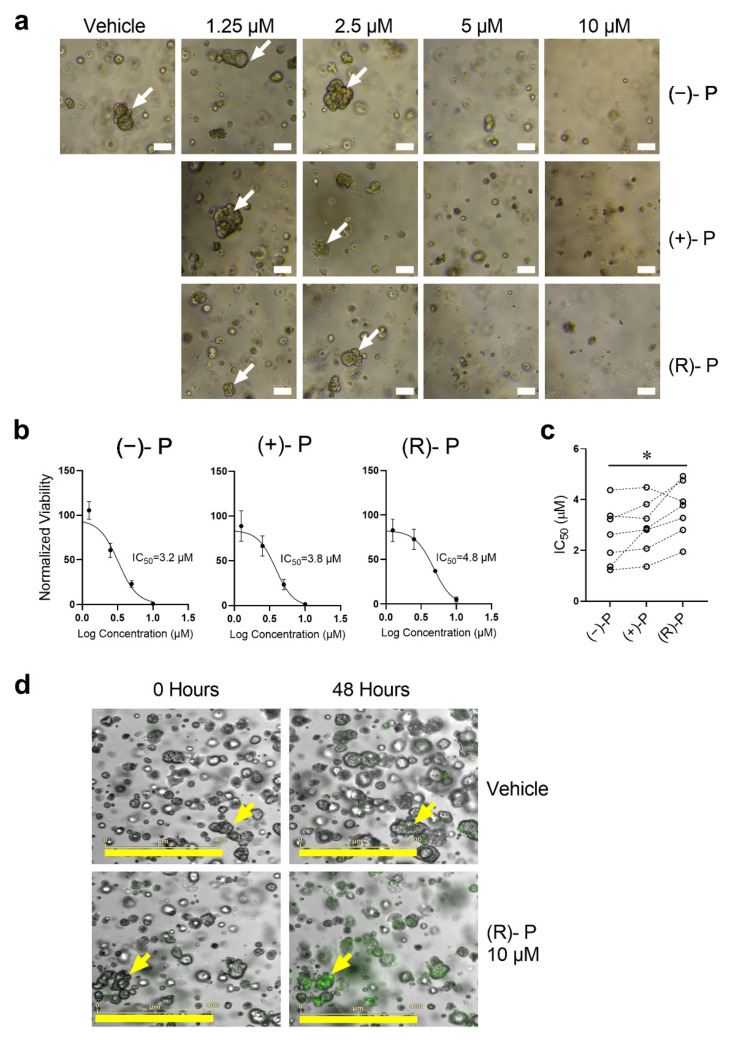
Disruption of CRC organoid structure following treatment with (−)-P, (+)-P or (R)-P. CRC organoids were grown for 48 h followed by treatment with (−)-P, (+)-P and (R)-P for 144 h. Images were taken before the addition of AlamarBlue dye. (**a**) Representative images of patient-derived liver metastatic CRC organoids (TQEH196) treated with (−)-P, (+)-P and (R)-P. White bars represent 100 µm. White arrows indicate viable organoids. (**b**) Growth curve and calculated IC_50_ values for (−)-P, (+)-P and (R)-P of TQEH196. Individual points represent mean ± SD from four technical replicates. (**c**) IC_50_ values for seven individual CRC patients. Dashed line denotes organoid IC_50_ responses from the same patient. * *p* ≤ 0.05, Friedman repeated measure ANOVA. (**d**) Representative images showing caspase 3/7 activation (green) denoting apoptosis by 10 μM of (R)-P in a patient-derived organoid 48 h post treatment. Yellow bars represent 400 µm; yellow arrows represent organoids tracked overtime.

Like the CRC cell line spheroid data, our study of seven patient-derived CRC organoids showed that treatment with perhexiline compounds reduced CRC organoid viability as evidenced by disruption of organoid structures (Figure 5a) and reduced viability as measured by AlamarBlue assay (Figure 5b). The IC50 values for (−)-P, (+)-P and (R)-P were similar to those measured in the CRC cell lines regardless of tumour location or DNA mismatch repair (MMR) status (Table 2 and Figure 5c). Interestingly, (−)-P had a marginally smaller IC50 when compared to (R)-P. Furthermore, we show that perhexiline induces apoptosis in these organoids, as confirmed by caspase 3/7 activation in one (R)-P treated CRC organoid (Figure 5d, Supplementary Videos S5 and S6). Finally, the dose–response curves of normal liver organoids show that perhexiline is better tolerated by normal tissue (Figure S3), consistent with our finding that normal HFF are less responsive to the drugs in vitro. Together, our data indicate that perhexiline and its enantiomers induce apoptosis and kill cancer cells in vitro.

## 4. Discussion

The results reported here suggest that perhexiline warrants further investigation as a novel, repurposed drug for use in the management of CRC. Perhexiline inhibited the growth of each of the five CRC cell lines tested, whether grown as monolayers or spheroids. It induced apoptosis in the cancer cells, as measured by an increase in annexin V induction by flow cytometry 48 h after treatment or by caspase 3/7 expression measured by live-cell imaging over 72 h. It reduced the viability of patient-derived organoids, as evidenced by the disruption of organoid structures. The individual enantiomers of perhexiline were each as effective as the racemic mixture, the form of the drug dispensed for clinical use.

In many cancers, including CRC, there are metabolic alterations that promote FAO, and these have been associated with increased tumorigenesis and metastasis. This has led to an interest in the possible use of FAO inhibitors in the management such cancers [17,18]. Perhexiline disrupts FAO by inhibiting mitochondrial uptake of long-chain fatty acids [5]. There have only been a few reports of the effect of perhexiline alone on cancer cells, all using the racemic mixture of the drug. Some of the studies concluded that perhexiline acted by mechanisms other than inhibition of FAO [19,20,21]. Perhexiline has been reported to kill human chronic lymphocytic leukemia cells in vitro and in a mouse model of the disease [22] and human T-cell acute lymphoblastic leukemic cells [23]. It was cytotoxic in vitro to a panel of glioblastoma cell lines and inhibited in vivo tumour growth in both flank and orthotopic glioblastoma models. Interestingly, perhexiline did not affect FAO in these models, and the authors concluded that its target was the protein tyrosine kinase oncogene FYN [20]. Perhexiline inhibited breast cancer cell proliferation in vitro and tumor growth in vivo by promoting selective HER3 receptor internalization and degradation. This inhibited HER3-mediated signaling, resulting in inhibition of breast cancer cell proliferation in vitro. Tumor growth was reduced in vivo when given as combination therapy with lapatinib, a tyrosine kinase inhibitor which can inhibit some types of breast cancer cell growth [9].

There are other reports as well which suggest that perhexiline in combination with conventional chemotherapy results in superior outcomes compared to chemotherapy alone. Early interest in the use of perhexiline in cancer arose from observations that it reversed acquired resistance to doxorubicin in murine leukemia [7] and breast cancer cells [8]. More recently, perhexiline has been reported to enhance the therapeutic efficacy of cisplatin in killing epithelial ovarian cancer cells in vitro and in an intraperitoneal xenograft model [24] and neuroblastoma cells in vitro and in vivo [13]. In a study of prostate cancer treatment, low doses of perhexiline only modestly inhibited proliferation of the LNCaP cell line in vitro, while combination treatment with perhexiline and either abiraterone or enzalutamide almost completely blocked proliferation [11].

There is only one report in the literature of the effect of perhexiline on CRC cell lines. Wang et al., in a study of gastrointestinal cancers, reported that perhexiline in combination with oxaliplatin suppressed the progression of gastrointestinal cancer cell lines in vitro and in mouse models [12]. We extend that report using more cell lines, caspase activation as a measure of apoptosis, and confirm the results using cultures of 3D cell line spheroids and patient-derived organoids.

Cancer cell lines derived from primary patient tumours and grown as 2D monolayers are widely used as preclinical cancer models for the screening of drugs and assessing responses to drugs. However, it is clear that the biochemical and genetic characteristics of cancer cell lines differ significantly from those found in primary tumours. As well, 2D cultures lack the tissue architecture and cell–cell or cell–matrix interactions found in tissues. It is perhaps unsurprising, then, that drugs which are effective against cell lines in 2D cultures are often ineffective in clinical trials. Three-dimensional culture systems are being more widely used, as findings show they better reflect what occurs in vivo [25,26]. Their utility has expanded from drug screens to include toxicity-induced cellular alterations [27], cancer invasion [28] and development of drug resistance [29]. In this study we measured the effect of perhexiline in two 3D culture systems, cell line spheroids and patient-derived organoids. These better replicate the architecture, microenvironment and growth conditions of cancer cells in vivo, and are considered preferable for drug studies. Patient-derived tumour organoids are considered the best in vitro model with which to discover and test novel anticancer drugs [13,14]. They are derived from pluripotent stem cells or isolated organ progenitors from the primary tumour that differentiate to form an organ-like tissue exhibiting multiple cell types. Organoids can self-renew and self-organize and they retain key structural, genomic and functional characteristics of the original tumour. Patient-derived organoid cultures have been recently shown to retain their tissue identity from both normal liver and colorectal cancer tissues [30,31]. Several studies have shown a correlation between the response to drugs by organoids in vitro and the source tumour in vivo.

Perhexiline has been used for decades for the treatment of cardiovascular diseases, but its use has been limited due to concern about hepatotoxicity and neurotoxicity which has been observed in a minority of patients. Toxicity is only observed after longer term use. It can be avoided by careful monitoring of perhexiline plasma concentrations and adjusting dosing to keep plasma concentrations below 0.6 mg/L (equivalent to ~2 µM) [5,19,32,33]. Compared to plasma, in vivo perhexiline tissue concentrations are markedly higher, as demonstrated in the livers and hearts of rats and humans [34,35,36]. Tumour clearance studies show that a daily oral regimen of up to 7 mg/kg (~10 µM) perhexiline for 4 weeks was well tolerated in mice and produced significant reduction in tumour sizes for different tumour types [12,20,22,37]. One aim of this study was to identify if there were any differences in the effects on cancer cells in vitro between the racemic form of perhexiline and its enantiomers. Previous pharmacokinetic studies showed significant enantioselectivity in perhexiline metabolism. The (−)-P was more readily metabolized and therefore cleared faster from the body than (+)-P [9]. This enantioselectivity was shown to be partially dependent on the expression of cytochrome P450 2D6 (CYP2D6) [38]. Expression of CYP2D6 may be altered by culturing cells as 3D spheroids [27], which could explain the differences in spheroid killing kinetics observed between the enantiomers. Nevertheless, we show that while there were subtle differences between the enantiomers, both ultimately inhibited cancer cell growth and induced cell death in vitro. Future mechanistic studies will be required to determine if the enantiomers elicit distinct changes to CRC cells.

There are limitations in this study that should be noted. While we have shown that perhexiline is cytotoxic to CRC cells, we have not investigated if this is due to CPT-1 inhibition or some other mechanism. Whilst all our cell lines and patient-derived organoids responded to perhexiline treatment, we have not determined if CRC cells have the potential to adapt to exposure to perhexiline and develop resistance. Furthermore, while both enantiomers are effective in inducing tumour cytotoxicity, in vivo investigations are required to determine potential enantioselective tissue localisations and drug clearance rates. This could suggest if there were a place in the clinical management of CRC for the use of one of the enantiomers rather than the racemate.

## 5. Conclusions

This is the most comprehensive study to assess the potential of perhexiline for use in the management of CRC. It was effective at killing CRC cell lines as either monolayers or spheroids and patient-derived organoids from primary and metastatic sites. We observed subtle enantioselective properties in tumour cytotoxicity that need to be elucidated with in vivo studies, including tumour clearance and tissue distribution. Our study is a promising first step towards the possible repurposing of perhexiline for application in the treatment of CRC.

## Figures and Tables

**Table 1:** The IC_50_ of (+)-P, (−)-P and (R)-P on cell lines tested expressed as mean ± SD (µM).

Cell Line	Cell Type	(−)-P	(+)-P	(R)-P
COLO205	CRC	4.27 ± 0.51	4.39 ± 0.47	3.29 ± 0.90
HCT116	CRC	3.37 ± 0.82	5.01 ± 1.91	3.45 ± 0.74
HT29	CRC	3.81 ± 1.00	4.25 ± 0.32	3.08 ± 0.41
SW480	CRC	3.70 ± 1.30	3.92 ± 0.74	3.92 ± 0.99
SW620	CRC	3.88 ± 0.48	4.30 ± 0.70	3.62 ± 0.91
HFF	Fibroblast	11.06 ± 1.04	12.04 ± 0.53	11.34 ± 0.85

**Table 2:** Selectivity index of (+)-P, (−)-P and (R)-P against HFFs.

Cell Line	(−)-P	(+)-P	(R)-P
COLO205	2.59	2.74	3.45
HCT116	3.28	2.40	3.29
HT29	2.90	2.83	3.68
SW480	2.99	3.07	2.89
SW620	2.85	2.80	3.13

**Table 3:** CRC patient-derived organoids—patient characteristics and IC_50_ of response to (−)-P, (+)-P and (R)-P.

Sample	Sex	Age	Location	Stage	Primary/Metastasis	MMR *	IC_50_ (µM)		
							(−)-P	(+)-P	(R)-P
TQEH 196	M	59	Liver	T4	Metastasis	MSS	3.23	3.82	4.76
TQEH 198	M	68	Liver	T4	Metastasis	MSS	1.36	2.87	3.78
SAH01	F	50	Lung	T4	Metastasis	MSS	1.23	1.36	1.95
RAH038	F	87	Colon (Ascending)	T3	Primary	MSI (Absent MLH1 and PMS2)	4.38	4.48	3.93
RAH057	F	65	Colon (Sigmoid)	T3	Primary	MSS	2.62	2.81	3.28
RAP05	M	71	Colon (Caecum)	T3	Primary	MSS	3.36	3.25	4.93
RAH51	F	82	Colon (Hepatic Flexure)	T4	Primary	MSI (Absent MLH1 and PMS2)	1.91	2.07	2.80

* Mismatch repair (MMR) status denoted either as: Microsatellite Stable (MSS) or Microsatellite Instable (MSI).

## Data Availability

The data presented in this study are available on request from the corresponding author. The data are not publicly available due to patient information confidentiality.

## Supplementary Materials

The following are available online at https://www.mdpi.com/article/10.3390/cancers14041043/s1, Figure S1: The caspase3/7 activation kinetics of (a) SW620 and (b) HCT116 CRC cell lines grown in monolayers for 72 h in the presence of (−)-P, (+)-P, and (R)-P and Caspase-3/7 Green reagent and monitored using an Incucyte S3 Live Analysis System. Figure S2: The kinetics of (−)-P, (+)-P, and (R)-P induced caspase 3/7 activation in HCT116 cells grown as spheroids. HCT116 spheroids were grown for 96 h, followed by live cell imaging for a further 96 h in the presence of (−)-P, (+)-P, and (R)-P and Caspase-3/7 Green reagent using an Incucyte S3 Live analysis system. Figure S3: Viability of normal liver organoids following treatment with (−)-P, (+)-P or (R)-P. Videos S1–S4: Supplementary videos in relation to Figure 4. Videos show time-lapse microscopy of HT29 spheroids treated with of (−)-P, (+)-P, and (R)-P and Caspase-3/7 Green reagent captured using an Incucyte S3 Live analysis system. Each video represents the following: Video S1: Vehicle Treatment; Video S2: 10 µM (−)-P; Video S3: 10 µM (+)-P; Video S4: 10 µM (R)-P, Videos S5 and S6: Supplementary videos in relation to Figure 5d. Videos show time-lapse microscopy of CRC organoids (TQEH 196) treated with 10 µM of (R)-P and Caspase-3/7 Green reagent captured using an Incucyte S3 Live analysis system. Each video represents the following: Video S5: 10 µM (+)-P; Video S6: Vehicle Treatment.
