# Supplementary material for: The Antianginal Drug Perhexiline Displays Cytotoxicity against Colorectal Cancer Cells In Vitro: A Potential for Drug Repurposing
Source: Cancers (Basel). 2022 Feb 18;14(4):1043. doi: 10.3390/cancers14041043 (PMC8869789; doi:10.3390/cancers14041043)
Supplement: Supplementary file 1 [file cancers-14-01043-s001.zip › Cancers-Dhakal-2022-Supplementary-Revised.pdf]

Supplementary Figures S1-S3

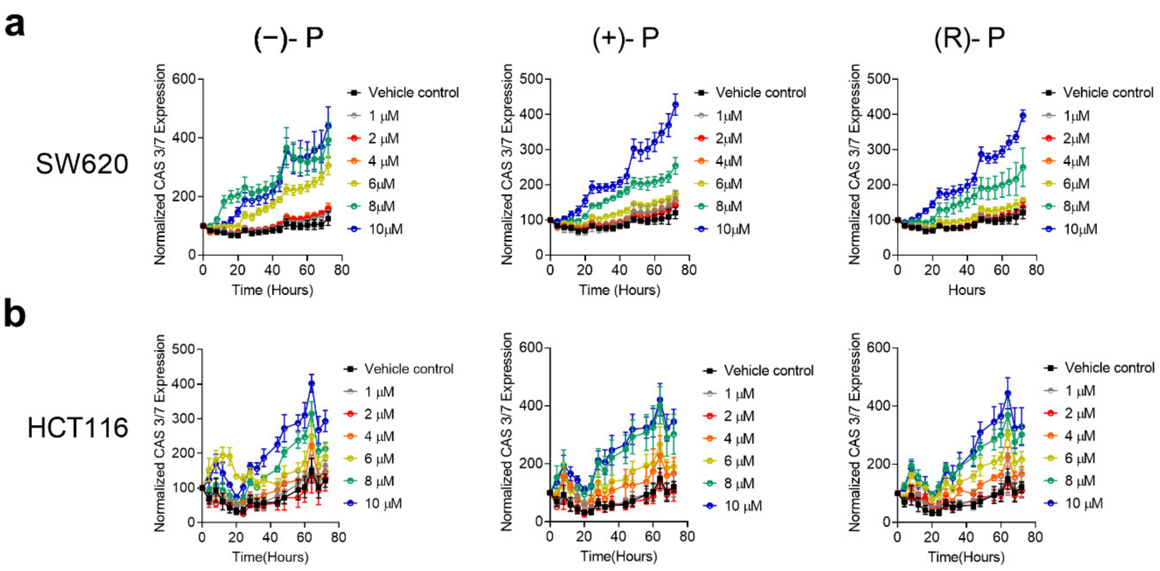

Figure S1. The caspase3/7 activation kinetics of (a) SW620 and (b) HCT116 CRC cell lines grown in monolayers for 72 hours in the presence of (−)-P, (+)-P, and (R)-P and Caspase-3/7 Green reagent and monitored using an Incucyte S3 Live Analysis System. The representative curves were generated by calculating the CAS 3/7+ (green) cells measured normalized to time 0. Individual points represent mean ± SD from three technical replicates. Data is representative of two independent experiments.

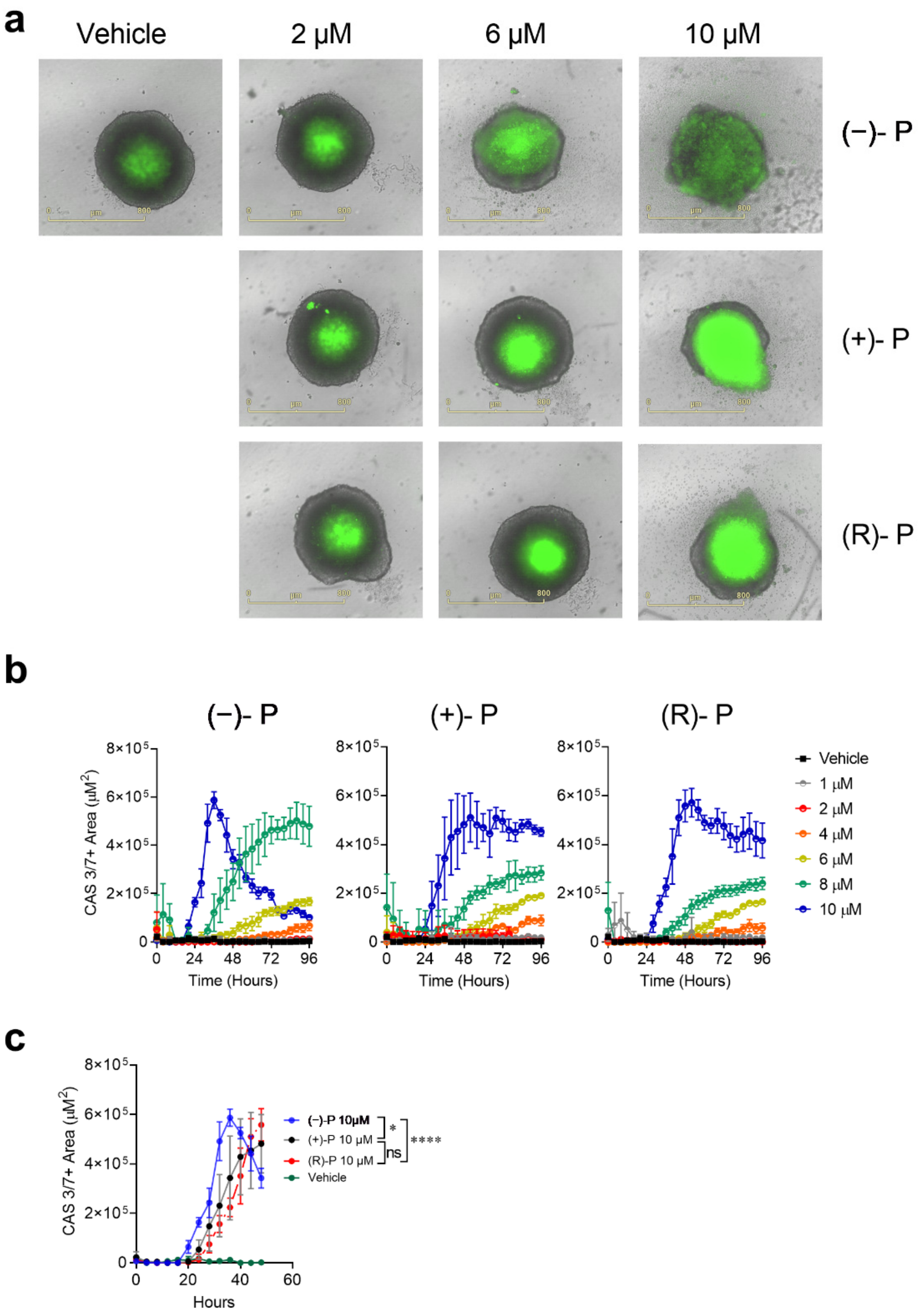

Figure S2. The kinetics of (−)-P, (+)-P, and (R)-P induced caspase 3/7 activation in HCT116 cells grown as spheroids. HCT116 spheroids were grown for 96 hours, followed by live cell imaging for a further 96 hours in the presence of (−)-P, (+)-P, and (R)-P and Caspase-3/7 Green reagent using an Incucyte S3 Live analysis system. (a) Representative images of HCT116 spheroids 96 hours post-drug treatment. Yellow bars represent 800 µm. (b) Caspase 3/7 kinetics curves generated by measuring the caspase 3/7 positive area within HCT116 spheroids for (−)-P, (+)-P, and (R)-P over 96 hours. (c) Comparison of caspase 3/7 activation over 48 hours after treatment with vehicle or 10 µM (−)-P, (+)-P and (R)-P. Individual points represent the mean ± SD of three technical replicates in a single experiment. * $p \leq 0.05$, **** $p \leq 0.0001$. Two-way ANOVA. Data is representative of two independent experiments.

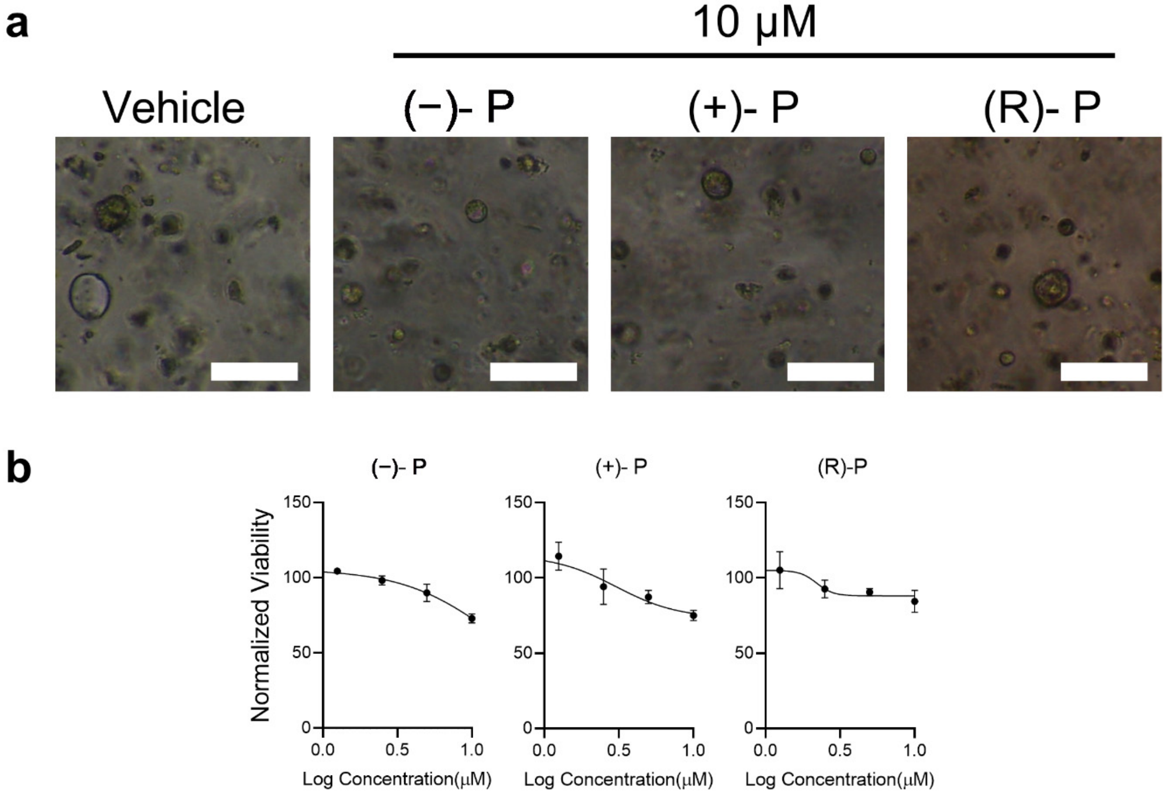

Figure S3. Viability of normal liver organoids following treatment with (−)-P, (+)-P or (R)-P. Normal liver organoids were grown for 48 hours followed by treatment with (−)-P, (+)-P and (R)-P for 144 hours. Images were taken before the addition of AlamarBlue dye. (a) Representative images of normal liver organoids treated with (−)-P, (+)-P, and (R)-P. White bars represent 50 µm. (b) Representative growth curves for normal liver organoids in the presence of (−)-P, (+)-P, or (R)-P. Mean ± SD with individual points representing four technical replicates from 1 patient.

Supplementary Videos S1-S4

Supplementary videos in relation to Figure 4. Videos show time-lapse microscopy of HT29 spheroids treated with of (−)-P, (+)-P, and (R)-P and Caspase-3/7 Green reagent captured using an Incucyte S3 Live analysis system. Each video represents the following: Supp Video S1 : Vehicle Treatment; Supp Video S2: 10 µM (−)-P ; Supp Video S3:10 µM (+)-P; Supp Video S4: 10 µM (R)-P .

Supplementary Videos S5 and S6

Supplementary videos in relation to Figure 5d. Videos show time-lapse microscopy of CRC organoids (TQEH 196) treated with 10 µM of (R)-P and Caspase-3/7 Green reagent captured using an Incucyte S3 Live analysis system. Each video represents the following: Supp Video S5: 10 µM (+)-P; Supp Video S6: Vehicle Treatment.
